# Identification of Novel Schizophrenia Loci by Homozygosity Mapping Using DNA Microarray Analysis

**DOI:** 10.1371/journal.pone.0020589

**Published:** 2011-05-31

**Authors:** Naohiro Kurotaki, Shinya Tasaki, Hiroyuki Mishima, Shinji Ono, Akira Imamura, Taeko Kikuchi, Nao Nishida, Katsushi Tokunaga, Koh-ichiro Yoshiura, Hiroki Ozawa

**Affiliations:** 1 Department of Neuropsychiatry, Nagasaki University Graduate School of Biomedical Sciences, Nagasaki, Japan; 2 Department of Human Genetics, Nagasaki University Graduate School of Biomedical Sciences, Nagasaki, Japan; 3 Nagasaki University Global Center of Excellence Program, Nagasaki, Japan; 4 Department of Human Genetics, Graduate School of Medicine, The University of Tokyo, Tokyo, Japan; Baylor College of Medicine, United States of America

## Abstract

The recent development of high-resolution DNA microarrays, in which hundreds of thousands of single nucleotide polymorphisms (SNPs) are genotyped, enables the rapid identification of susceptibility genes for complex diseases. Clusters of these SNPs may show runs of homozygosity (ROHs) that can be analyzed for association with disease. An analysis of patients whose parents were first cousins enables the search for autozygous segments in their offspring. Here, using the Affymetrix® Genome-Wide Human SNP Array 5.0 to determine ROHs, we genotyped 9 individuals with schizophrenia (SCZ) whose parents were first cousins. We identified overlapping ROHs on chromosomes 1, 3, 4, 5, 6, 7, 8, 9, 10, 11, 12, 13, 16, 17, 19, 20, and 21 in at least 3 individuals. Only the locus on chromosome 5 has been reported previously. The ROHs on chromosome 5q23.3–q31.1 include the candidate genes histidine triad nucleotide binding protein 1 (*HINT1*) and acyl-CoA synthetase long-chain family member 6 (*ACSL6*). Other overlapping ROHs may contain novel rare recessive variants that affect SCZ specifically in our samples, given the highly heterozygous nature of SCZ. Analysis of patients whose parents are first cousins may provide new insights for the genetic analysis of psychiatric diseases.

## Introduction

Schizophrenia (SCZ) is categorized as a severe chronic debilitating psychosis that affects approximately 1% of the global population. Although genetic factors are reported to contribute to the disease and multiple responsible loci have been identified from linkage analysis and case-control association studies, there have been few reproducible results to date [1].

Morrow et al. (2008) [2] suggested that homozygosity mapping is a powerful tool not only for investigating single gene defects but also for rare genomic variants in complex traits. They observed homozygous deletions in patients with autistic disorders and concluded that genomic alterations might be a subset of disease-causing mutations in chromosomal regions. The increased susceptibility to SCZ observed in consanguineous families suggests that genomic recessive variations may be involved in its etiology. [3]–[5] Considering this and other results, we hypothesized that homozygosity mapping, including identical by descent (IBD) analysis, would be a highly constructive method for identifying the loci responsible for SCZ.

We hypothesized that runs of homozygosity (ROHs) could contribute to SCZ by a recessive effect. We use the term “ROH” [6] instead of loss of heterozygosity (LOH) for regions where homozygous genotypes are contiguous because LOH implies heterozygous deletions or hemizygosity, while ROH suggests consecutive homozygous regions. Recessive effects are obtained by genetic variations including single nucleotide variations, small insertions/deletions, structural variations, and chromosomal rearrangements. These variations may affect amino acid sequences or the control of gene expression, including small RNA expression.

**Figure 1:**
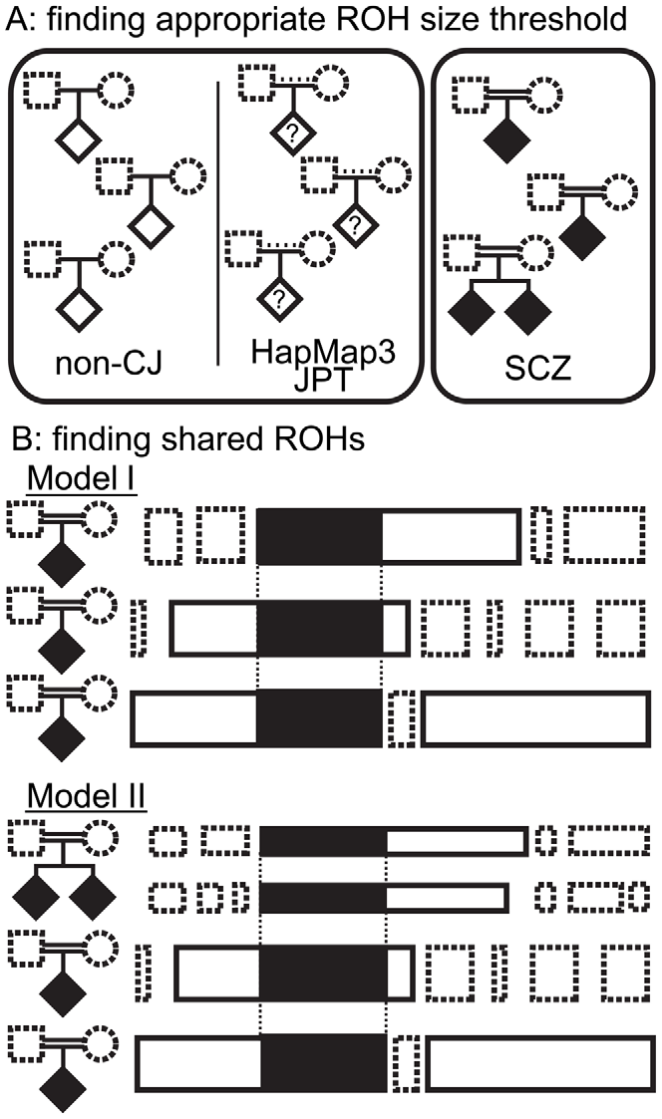
Two-stage design of this study. A, the first stage was to find an appropriate autosomal run of homozygosity (ROH) size threshold to distinguish specific ROHs from the offspring of first-cousin marriages from ROHs in the offspring of non-consanguineous marriages. The size distribution of ROHs in our non-consanguineous Japanese (non-CJ) and schizophrenia (SCZ) samples was compared. Non-CJ samples are the offspring of non-consanguineous marriages that were validated by interview. Here, SCZ samples were used as the offspring of first-cousin marriages regardless of phenotype. Samples from parents were not used in this study (dashed squares and circles). To confirm our strategy, we also assessed HapMap3 JPT samples, which do not have information for phenotypes or family consanguinity (dashed and solid lines between parents). B, the second stage was to find shared ROHs among the SCZ samples as patients with schizophrenia. In Model I, an autosomal ROH size threshold was applied to filter out smaller ROHs (dashed open boxes). Larger ROHs (solid open boxes) were assessed to find overlaps among patients (solid boxes). In Model II, after filtering by the ROH size threshold, ROHs shared by the siblings of patients and ROHs of other patients were assessed to find overlaps. In this study, the gender of the samples was not matched (diamonds) because we only evaluated autosomal ROHs.

Here, we describe a homozygosity mapping strategy that consisted of 2 stages (Figure 1). The first stage aimed to find the appropriate size threshold for autosomal ROHs that would distinguish ROHs specifically existing in the offspring of first-cousin marriages from those that commonly exist in the offspring of non-consanguineous marriages. By comparing the size distribution of ROHs between the offspring of first-cousin marriages and non-consanguineous marriages, we concluded that ROHs >2.1 Mb in size in the offspring of consanguineous marriages can be assumed to be IBD segments from an individual 3 generations before. The second stage aimed to find shared ROHs among patient with SCZ using 2 models. In Model I, an autosomal ROH size threshold was applied to filter out smaller ROHs. Larger ROHs were assessed to find overlaps among the patients. In Model II, after filtering by the ROH size threshold, ROHs shared by the siblings of patients and ROHs of other patients were assessed to find overlaps. The overlapping ROHs we identified potentially contain SCZ causative regions that are specific to our samples because of the heterogeneous nature of SCZ.

## Materials and Methods

### 1. Samples

A total of 9 subjects with SCZ (3 males and 6 females, aged 31–56 years) (SCZ individuals) were recruited to this study after being diagnosed as having typical paranoid schizophrenia by a certified psychiatrist (N.K.) using the *Diagnostic and Statistical Manual of Mental Disorders*, Fourth Edition, Text Revision (DSM-IV-TR) and the *Structured Clinical Interview for DSM-IV Axis I Disorders* (SCID). The study received ethics approval from the Committee for Ethical Issues on Human Genome and Gene Analysis at Nagasaki University, Japan. All of the patients were from the main islands of Japan, excluding Okinawa. We obtained written informed consent from all participants. The consanguineous patients were from 8 first-cousin marriages. Seven individuals (patients a to g) were unrelated and 2 were siblings (patients h-1 and h-2). We also recruited 92 healthy individuals from non-consanguineous marriages (non-CJ individuals) from the main islands of Japan, excluding Okinawa. We confirmed consanguinity by interview. We did not match for gender in the SCZ and non-CJ individuals because we only intended to analyze autosomal chromosomes.

After obtaining written informed consent, genomic DNA was isolated from peripheral blood. We did not collect blood samples from the patients' parents, except for 1 patient, or siblings; however, we confirmed that they had no history of psychiatric illness, with the exception of the older brother of patient g, by direct interview or from the medical records of the other related individuals.

Furthermore, we also assessed the International HapMap Project [7] phase 3 data of the Japanese in Tokyo (HapMap3 JPT) to evaluate the non-CJ individuals. Raw signal intensity files (CEL files) obtained using Affymetrix Genome-Wide Human SNP Array 6.0 (Affy6.0) were downloaded from http://www.hapmap.org/.

### 2. Microarray analysis

We performed genome-wide SNP genotyping of 9 SCZ samples and 92 non-CJ samples using the Affymetrix Genome-Wide Human SNP Array 5.0 (Affy5.0) according to the manufacturer's instructions. Our microarray data is MIAME compliant and the raw data has been deposited in the CIBEX database (CIBEX accession number: CBX141).

### 3. ROH detection

We generated the CHP genotype files from the CEL signal intensity files using the BRLMM-P genotype calling program [8], [9]. For the detection of ROHs, we analyzed the CHP files with a hidden Markov model (HMM)-based ROH detection function of the Partek® Genomics Suite (Partek GS) software version 6.5 build 6.11.0207 (Partek, St. Louis, MO, USA). We applied the following default HMM parameters: max probability = 0.99, genomic decay = 0 (disabled), genotype error = 0.01, and default frequency = 0.3. We did not adopt the baseline files.

**Figure 2:**
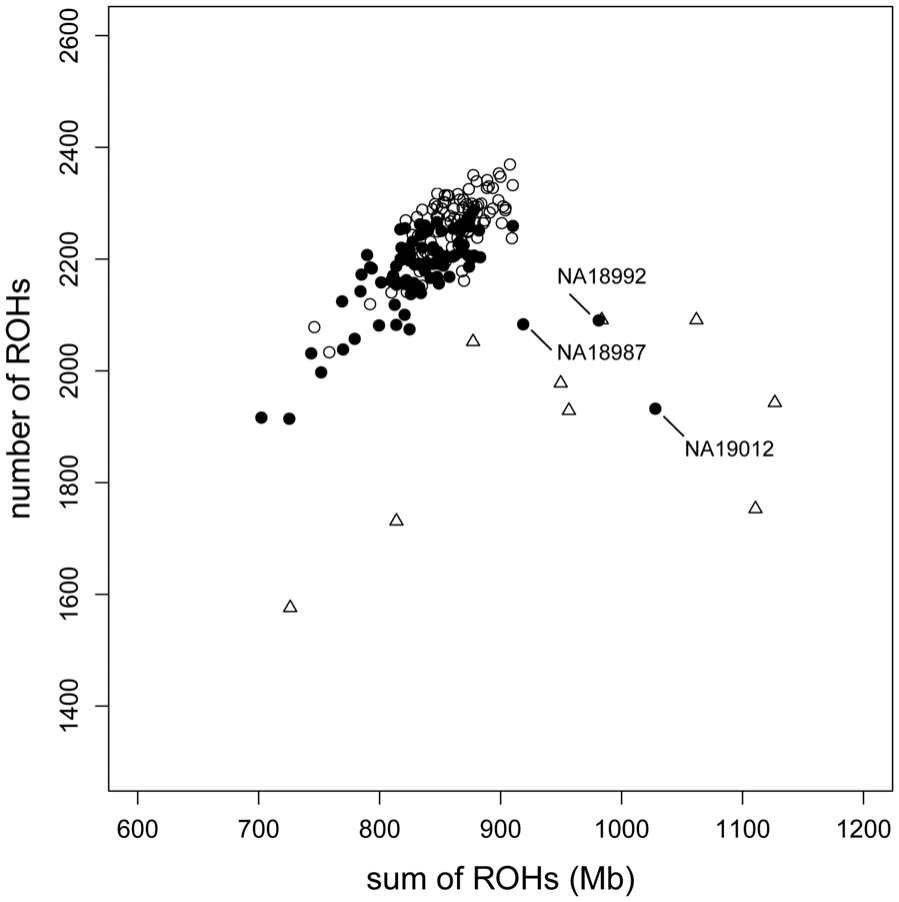
Distribution of the size and number of individual autosomal runs of homozygosity (ROHs). Sums and total numbers of individual ROHs are shown by circles and triangles indicating unrelated Japanese individuals (non-CJ: 92 samples) and the offspring of first-cousin marriages with schizophrenia (SCZ: 9 samples), respectively.

**Figure 3:**
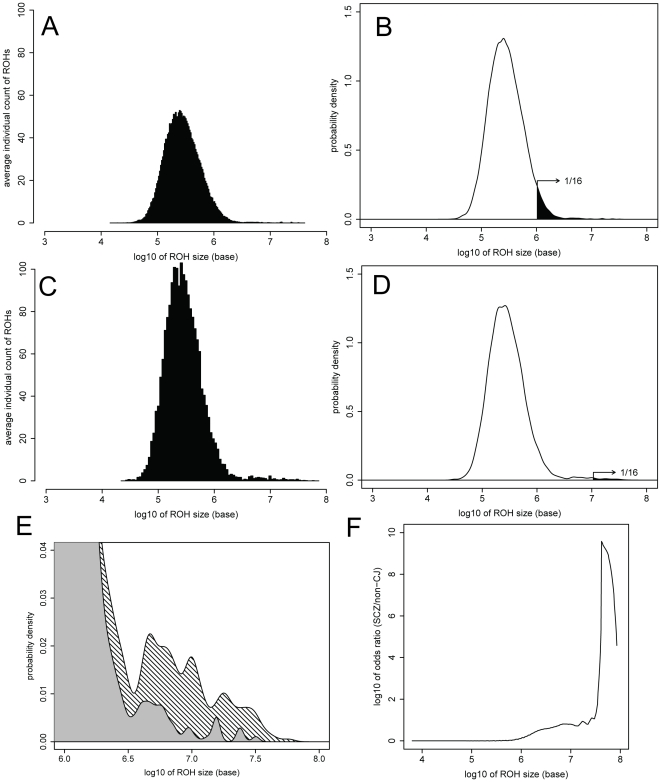
Size distribution of autosomal runs of homozygosity (ROHs). In the size distribution plot of non-consanguineous Japanese (non-CJ; A and B) and schizophrenia (SCZ; C and D) samples, the x-axis indicates the ROH size (log_10_ scale). A and C, individual average frequency of the ROHs as histograms. B and D, estimated probability density corresponding to each histogram. Black areas shows 1/16 (6.25%) of autosomes, which is equivalent to the expected sum of autozygous regions in the offspring of a first-cousin marriage. E, enlarged overlap of B (gray) and D (hatched). F, SCZ/non-CJ odds ratio plot. X-axis indicates the size of the ROHs (log_10_ scale). Y-axis (log_10_ scale) indicates the ratio of areas exceeding the given ROH size threshold in the estimated probability distributions of the SCZ and non-CJ datasets.

Detected ROHs were statistically analyzed and visualized (Figures 2 and 3; Tables 1 and 2) by using in-house scripts written in the R language [10]. The optimization of histogram bandwidths and the estimation of the probability density distributions were performed using the “KernSmooth” package of R [11].

**Table 1:** Autosomal runs of homozygosity (ROHs) size distribution, where descriptive statistics of ROH sizes were detected with Partek GS.

Dataset	N	Minimum^a^	Mode^b^	Maximum^c^	Average sum^d^
HM3JPT^e^	88	19 750 (14)	256 499 (27)	32 000 000 (1252)	831 159 144
non-CJ^f^	92	18 160 (14)	248 288 (27)	32 250 000 (1921)	859 784 793
SCZ^g^	9	27 380 (14)	258 488 (38)	57 810 000 (9896)	956 266 858

a Minimum ROH size in all individuals from each dataset.

b Mode ROH size in all individuals from each dataset.

c Maximum ROH size in all individuals from each dataset.

d Average sum is the average total ROH size per individual from each dataset.

e The International HapMap Project phase 3 Japanese in Tokyo. Three samples, NA18987, NA18992, and NA19012, of 91 samples are omitted because they are potentially the offspring of a consanguineous marriage.

f Non-consanguineous Japanese.

g Schizophrenia.

Numbers are in bases, and the numbers in parentheses are the included probe sets.

**Table 2:** Thresholds, individual average sums of runs of homozygosity (ROHs), its ratio in the autosomal genome, and the individual average encompassed number of ROHs corresponding to the odds ratios.

Odds ratio	Threshold (base)	Non-CJ^a^ dataset			SCZ^b^ dataset		
		sum (base)	Autosomal ratio (%)	# of ROHs	sum (base)	Autosomal ratio (%)	# of ROHs
1.3	1 000 000	185 411 092	6.5	93.2	420 200 807	14.7	123.6
2.000	1 548 817	110 468 918	3.9	30.6	341 258 405	11.9	52.7
3.0	2 137 962	81 383 855	2.8	13.8	309 296 125	10.8	33.8
4.0	3 630 781	65 633 075	2.3	7.6	288 028 919	10.0	25.4
5.0	5 128 614	53 423 627	1.9	4.7	263 695 116	9.2	19.8
10.0	24 547 089	7 925 263	0.3	0.3	85 167 811	3.0	2.7

a Non-consanguineous Japanese.

b Schizophrenia.

Furthermore, to validate the data quality of our non-CJ samples, we also compared our data to HapMap3 JPT. Affy6.0 raw signal intensity data in CEL files were subjected to allele calling using Birdseed software version 2 [12]. SNP genotypes of shared loci between Affy6.0 and Affy5.0 were extracted and processed as well as the non-CJ and SCZ datasets to detect ROHs.

### 4. Detection of potential genetic loci for SCZ by overlapping ROHs

To detect the overlapping ROHs among the SCZ dataset, the identified ROHs were filtered by a size threshold on Partek GS, analyzed using an in-house Ruby script (available on request) to generate a table of overlapping ROHs, and visualized with Partek GS. Then, we extracted the loci shared among more than 3 unrelated individuals (Model I) (Table S1). Furthermore, on the basis of the hypothesis that concordant sibling cases share causal loci, we detected the loci shared among 2 sibling cases (h-1 and h-2) (Model II) and found the ROHs that were shared by 1 or more of the unrelated samples (Table 3).

**Table 3:** Novel loci identified in this study that are different from those in Table S1, for the segments overlapping in more than 1 unrelated individual and the common regions between the 2 siblings (cases h-1 and h-2).

Chromosome	Start	End	Samples	# Samples^a^	Length	Cytoband
1	146258078	148749860	h-1, h-2, a	3	2491783	1q21.1-q21.2
5	45437574	49631829	h-1, h-2, d	3	4194256	5p12-q11.1
5	117360252	120214932	h-1, h-2, f	3	2854681	5q23.1
5	120214932	122586267	h-1, h-2, f, g	4	2371336	5q23.1-23.2
7	57594442	62282881	h-1, h-2, b, f	4	4688440	7p11.2-q11.21
8	129121122	131617749	h-1, h-2, b	3	2496628	8q24.21-q24.22
8	132434559	139244531	h-1, h-2, b	3	6809973	8q24.22-24.23
10	37363792	37599485	h-1, h-2, e	3	235694	10p11.21
10	37599485	37874740	h-1, h-2, e, g	4	275256	10p11.21
10	37874740	42217616	h-1, h-2, c, e, g	5	4342877	10p11.21-q11.21
12	33982292	36255461	h-1, h-2, a, d	4	2273170	12p11.1-q11
13	35366458	43580724	h-1, h-2, g	3	8214267	13q13.3-14.11
16	28924029	29606107	h-1, h-2, c	3	682079	16p11.2
16	29606107	29657036	h-1, h-2, c, f	4	50930	16p11.2
16	29657036	29680943	h-1, h-2, c, d, f	5	23908	16p11.2
16	29680943	31277953	h-1, h-2, b, c, d, f	6	1597011	16p11.2
16	34467305	34647935	h-1, h-2, a, b, c, d, f, g	8	180631	16p11.1
16	34647935	45122807	h-1, h-2, a, c, d, f, g	7	10474873	16p11.1-q11.2
16	45122807	47094922	h-1, h-2, a, b, c, d, f, g	8	1972116	16q11.2-q12.1
17	29659797	32811528	h-1, h-2, a	3	3151732	17q12
19	37676724	40349191	h-1, h-2, a	3	2672468	19q13.11-13.12
21	19821557	20188026	h-1, h-2, g	3	366470	21q21.2

a Number of individuals (including h-1 and h-2) who shared the region; for example, 5 indicates that 3 other individuals shared the common region of the 2 siblings.

## Results

### 1. Determination of the ROH size threshold discriminating the offspring from non-consanguineous and first-cousin marriages

We genotyped 440 794 SNPs in each individual. Genotype calling rates for each sample ranged from 97.23–98.83% and their call rates were high and accurate enough for their subsequent evaluation. We utilized the data from 92 non-CJ and 91 HapMap3 JPT samples in addition to the data from 9 SCZ individuals.

Our homozygosity mapping strategy utilized differences in the length distribution of ROHs between offspring from consanguineous and non-consanguineous marriages. Individuals from consanguineous families are expected to have an increased number of longer ROHs containing autozygous segments. These segments were also expected to be discriminated by their length from ROHs containing homozygous segments by chance or by linkage disequilibrium (LD). To demonstrate the strategy, we performed detailed comparisons of the length distribution of ROHs between the non-CJ, HapMap3 JPT, and SCZ datasets.

We initially plotted the total number and size of ROHs in the non-CJ, HapMap3 JPT, and SCZ datasets (Figure 2). The non-CJ and HapMap3 JPT datasets clustered together, except for 3 individuals in HapMap JPT. These 3 outlier individuals, NA18987, NA18992 [13], and NA19012 [14], have been assumed to be from consanguineous families; indeed, the distribution of these samples was similar to that of our offspring from first-cousin marriages (Figure 2).

We then analyzed the length distribution of ROHs in the non-CJ and SCZ datasets. Bar plot histograms of the length of ROHs were obtained and the probability density curves were estimated by the “KernSmooth” package in R (Figure 3A–D). Descriptive statistics of these plots are also shown in Table 1. Both datasets produced bell curve-like distributions in the log_10_ scale on the x-axis to indicate the length of each ROH; however, the SCZ dataset showed a secondary peak in the larger ROH region. We expected that the autozygous region from the founders of the third ancestral generation (great-grandparents) would be larger in the SCZ dataset than in the non-CJ dataset, in whom LD may encompass ROHs by chance. The proportion of larger ROHs in the SCZ dataset was clearly higher than in the non-CJ dataset. As we can expect that 1/16 of the whole genome in the offspring of first-cousin marriages would be autozygous regions from their great-grandparents, we highlighted the graphs in Figure 3B and 3D at the point where the total sum of length in the upper tail of the ROH distribution reaches 179.2 Mb, which is 1/16 of the 2 867 732 772 bases total size of the autosomal haploid genome, according to the statistics from the NCBI Build 36.1 assembly (2006) [16]. This analysis suggested that it is highly probable that the longer ROHs would be inherited from the great-grandparents; however, it should be mentioned that genomic regions with less recombination tend to have longer ROHs.

To show further differences in the probability density distribution of the SCZ and non-CJ individuals, we also plotted an SCZ/non-CJ odds ratio (OR) plot (Figure 3F and Table 2), which indicates the ratio of probability for the existence of ROHs in each dataset over a given threshold length. To determine the overlapping ROH regions shared among the SCZ dataset, we adopted OR = 3.0 and the corresponding threshold of 2 137 962 bases to ensure practical power and to detect smaller IBD regions by recombination.

### 2. Determination of potential SCZ genetic loci by overlapping ROHs

**Figure 4:**
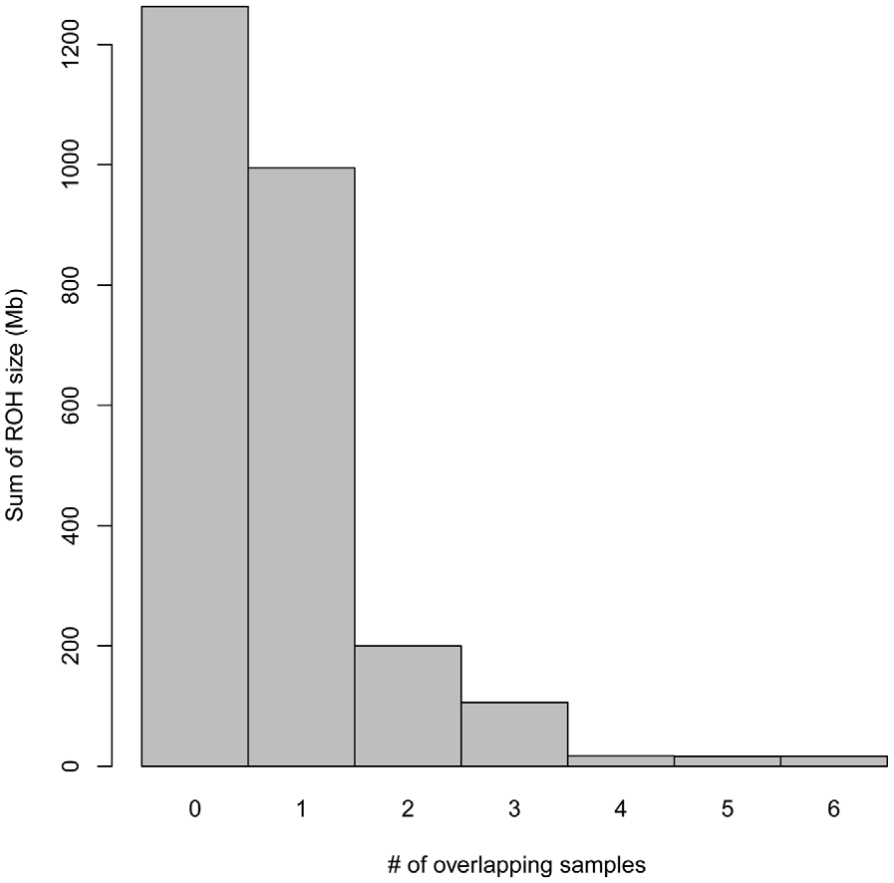
Sum of run of homozygosity (ROH) lengths and number of overlapping patients, excluding patient siblings. Y-axis indicates the sum of ROH lengths shared by a given number of patients. The zero column indicates the sum of ROHs not shared by any of the samples.

**Figure 5:**
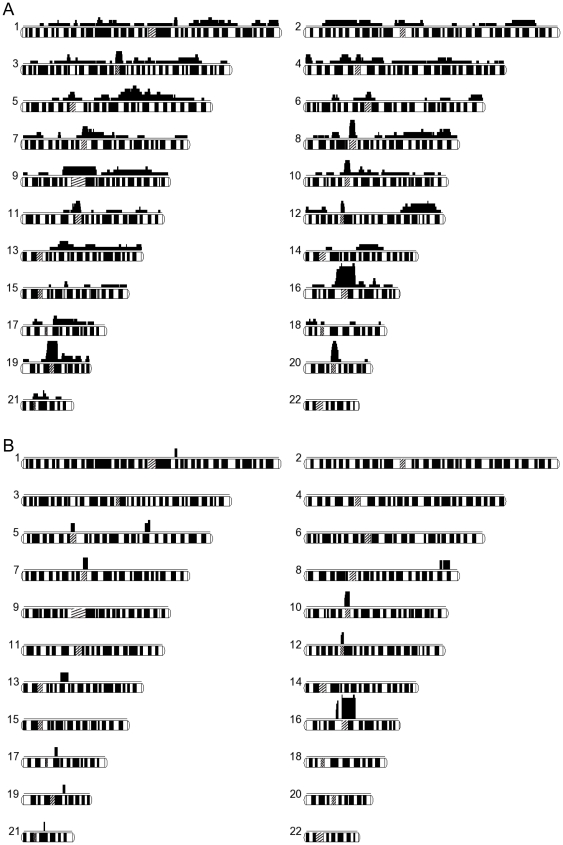
Overlapping autosomal runs of homozygosity. Each autosome is shown horizontally with the number of overlapping samples (upper) and chromosome ideograms (lower). Centromeres are shown by hatched boxes. A, overlapping segments shared among 1 (isolated) to 7 samples in a total of 9 patient samples. B, overlapping segments shared by 2 patient siblings (h-I and h-II) and an additional 1–4 patient samples.

The sum lengths of the overlapping regions among 0–7 independent family patients are shown in Figure 4, and the calculated percentage sum length among a given number of patients and more in the autosomal genome were as follows: 100%, 51.7%, 13.6%, 6.0%, 1.9%, 1.3%, and 0.6%. Considering the statistics, we adopted a minimum of 3 patients for identifying candidate loci. Figure 5 shows a schema of the overlapping ROHs within autosomes and their positions are summarized in Table S1.

Overlapping ROHs found in 3 or more SCZ individuals on chromosomes 1, 3, 4, 5, 6, 7, 8, 9, 10, 11, 12, 13, 16, 17, 19, 20, and 21 (Figure 5A) suggested that many loci are potentially associated with SCZ in our patients. Only the locus on chromosome 5 has been reported in a previous linkage analysis of SCZ [16]. The ROHs were expanded by the analysis of 4 additional individuals; however, no additional loci were detected (data not shown). The locus on chromosome 5q23.3–q31.1 included the regions containing the histidine triad nucleotide binding protein 1 (*HINT1*) and acyl-CoA synthetase long-chain family member 6 (*ACSL6*) genes. Our results suggest that recessive variants of these candidate genes could be involved in the pathogenesis of SCZ in our patients.

In the analysis of 2 siblings (h-1 and h-2) from a first-cousin marriage, we searched for the ROH regions shared by the siblings as a single gene defect. The detection of loci shared by the siblings and 1 or more unrelated individuals demonstrated ROHs on chromosomes 1, 5, 7, 8, 10, 12, 13, 16, 17, 19, and 21 that might be causative for SCZ (Figure 5B). Those loci did not include any previously reported candidate genes. Interestingly, among the loci detected in Figure 5A and 5B, there were no overlapping loci identified in this study.

## Discussion

### 1. Samples

We recruited 9 offspring from first-cousin marriages (SCZ) and 92 from non-consanguineous marriages (non-CJ). As shown in Figure 2, our non-CJ dataset and publicly available HapMap3 JPT datasets showed a common cluster, except for the presence of 3 outliers that have been reported to be potentially from consanguineous families [14], [15]. This concordance suggests that our experimental quality and data processing approaches were appropriate. In this study, we analyzed a limited number of samples; however, homozygosity mapping was a reasonable strategy to adopt because it requires relatively smaller number of samples than case-control studies. We did not use samples from the parents of patients in this study because these are not very informative in our strategy. On the other hand, affected and unaffected siblings in single families are strongly informative in homozygosity mapping, and we are continuously recruiting additional siblings for future study.

### 2. ROH analysis

Most of the previous homozygosity mapping studies were based on genotypes derived from microsatellites or simple tandem-repeat polymorphisms (STRP). The highly polymorphic nature of multiallelic STRP markers is suitable to cover the whole genome with a fewer numbers of markers. However, recent DNA microarray technologies have enabled massive genome-wide SNP genotyping to be performed in a short time. The problem with homozygosity mapping based on SNPs is the accurate detection of regions with ROHs. As SNPs have a less informative biallelic nature, using the naïve definition of an ROH as just a contiguous homozygous region may skew the detection of ROHs because of the frequency of low minor allele SNPs, genotyping errors, and “no-call” SNPs.

The solution to this problem using the Affymetrix Human Genotyping 500K arrays and Illumina Infinium HumanHap300v2 arrays was the application of ROH detection bins sliding through each chromosome to filter out low SNP density bins and to allow the small number of heterozygous SNPs and no-call SNPs to be placed in a bin [6], [17]. An alternative method to detect ROHs is to adopt an HMM. Partek GS software implements the HMM-based “LOH detection” algorithm. A similar algorithm is also implemented in the Affymetrix GeneChip Chromosome Copy Number Analysis Tool (CNAT), as described in the CNAT user guide [18]. The HMM-based algorithm of these tools takes not only the information of adjacent SNP genotypes but also the heterozygosity of SNPs as a reference baseline calculated from the genotyping results in the reference samples or the *a priori* default frequency. This method is expected to more accurately detect ROH regions that reflect actual recombination.

Selection of the reference population for the baseline data is crucial for the HMM-based detection of ROHs. If the reference population is carefully selected to match the background of the case population, the baseline generated from the observation of actual SNPs in the reference population can omit ROHs resulting from LD and regions with low SNP density, such as centromeres. However, if strict matching of the used population background is difficult, use of the fixed default heterozygous frequency, whose default value is 0.3, still has the advantage of minimizing false-negatives in the detection of ROHs.

To determine the optimal length threshold of ROHs to extract autozygous segments from whole ROHs, we adopted OR = 3 for the analysis. This approach may work well when a large enough reference sample is available. When a reference population is not available, a threshold where the sum of the ROH length in the upper tail of its distribution is equal to the theoretical autozygous length of a genome, that is, 1/16 of a genome in the offspring of a first-cousin marriage, could be another option. In our SCZ dataset, the threshold using this approach was approximately 10.6 Mb.

Our results demonstrated obvious differences in the proportion of the length distribution of ROHs between the non-CJ and SCZ datasets. A recent report on European populations, including endogamy subpopulations, has shown that a higher proportion of individuals in endogamy subpopulations have ROHs longer than 1.5 Mb compared with other subpopulations [6]. Our scatter plot of the individual total number and size of ROHs (Figure 2) is not fully in agreement with this previous report, although the non-CJ dataset made a cluster and showed a positive correlation (Pearson product-moment correlation coefficient r = 0.773), and the SCZ dataset was scattered and showed a weak positive correlation (r = 0.432). This may be explained by the fact that the previous report excluded ROHs <500 kb to ignore ROHs that potentially resulted from LD and removed hemizygous deletions of ROHs. In this study, we did not adopt a strategy to filter ROHs by their size before the analyses because the discrimination of autozygous regions and LD simply by size is essentially impossible. Adopting a baseline file derived from a strictly matched population in the HMM-based detection of ROHs can be used instead. Additionally, differences in genotyping platforms with different SNP densities may affect the size distribution of ROHs. Although our data from the sparser Affymetrix Genotyping 10k SNP panel produced a similar bell curve-like ROH size distribution, the whole curve was shifted to the right (data not shown).

The size distribution of ROHs for a given population is affected by its inbreeding coefficient (F). Studies of consanguineous marriages in subpopulations from Japan during the 1980s compared the F values for Japan (F = 0.00134) to those in Kuwait (F = 0.0219), India (F = 0.02313), England (F = 0.00017), and the United States (F = 0.00003) [19], [20]. These reports have also shown that despite the decrease in consanguineous marriages in Japan, local subpopulations have higher F-values. The same tendency has also been shown by a genealogical study that estimated inbreeding rates in large and semi-isolated populations on the basis of historical changes in population size [21]. Recently, the importance of studying endogamous populations has been stressed [22]; however, populations with intermediate F-values have advantages for our homozygosity mapping approach. This approach uses the differences in the size distribution of ROHs in a case population consisting of offspring from consanguineous marriages and a control population consisting of offspring from non-consanguineous marriages. A high F-value population may not have clear distribution differences between cases and controls. On the other hand, finding a sufficient number of cases in low F-value populations may not be easy. From this standpoint, an intermediate F-value population, such as the Japanese population, represents an interesting dataset for our homozygosity mapping approach, as was shown previously in the Costa Rican population [23], [24].

We presented here some threshold lengths of ROHs to detect IBD regions from great-grandparents. As recombination will, of course, occur everywhere by chance, small autozygous regions could be overlooked with the threshold shown here. However, no systematic analyses have so far identified IBD regions in consanguineous marriages by whole-genome SNP typing. Our method shown here, which 1) detects longer ROHs in each individual and 2) aligns ROHs and identifies overlapping regions, will be helpful for autosomal recessive disorders and also for complex disorders resulting from rare variants. If collecting patients in geographically and historically isolated areas is possible, this homozygosity mapping approach is likely to be successful. Nonetheless, the effectiveness of homozygosity mapping for complex disorders remains controversial [4]. We believe that we can uncover new candidate loci through the application of whole-genome SNP typing to homozygosity mapping because of its high density genomic coverage and high-throughput ability.

### 3. Possible novel loci for schizophrenia

We identified several putative SCZ loci that are presented in Figure 5 and Tables S1 and 3. In our study, we assumed the 2 models outlined in the Methods section. Model I was designed to find shared causal loci among unrelated individuals. For the other model, we hypothesized that the siblings shared the same causal loci; thus, Model II was designed to find the common loci between the siblings and unrelated individuals.

For Model I (Table S1), from the analysis of 7 unrelated individuals, the loci included the 5q23.3–q31.1 region that was previously identified by linkage analysis in the Irish population [16]. Among the genes that mapped to 5q23.3–q31.1, *HINT1* [25], [26] and *ACSL6* [27] were previously reported to be possibly associated with SCZ. In patients from consanguineous families we analyzed, homozygous genomic variations may be causative for the disease (Figure 5 and Tables S1 and 3). However, small sample size in our study may be a limiting factor to generalize such conclusions. In common diseases such as psychiatric disorders, including SCZ and bipolar disorders, especially in familial cases or in cases from relatively isolated areas, rare variants possibly contribute more than common variants to the disease phenotype [28]–[30].

On the basis of the rare variant-common disease hypothesis, it is appropriate that the genetic etiology between sibling cases and other unrelated cases may be various. In addition, our results suggested that multiple loci influenced the susceptibility to SCZ, as other reports have suggested [31].

We presented here the systematic analyses of the homozygosity mapping method using whole genome SNP typing, and we identified ROHs that potentially contain SCZ causative recessive regions that are shared among our samples. When we explain SCZ as a result of the homozygous state of rare variant mutations, the number of overlapping individuals may be challenging, as it is possible that each individual has a different variation. The heterogeneity of SCZ may explain the lack of overlap for our results with previously reported regions [32], [33]; moreover, our methodology has a limitation for detecting causative genes that are included in shorter ROHs by chance. We have shown that the Affymetrix Genome-Wide Human SNP Array 5.0 or 6.0 could be applied to special cases including first-cousin marriages to identify genomic variations. Increasing number of samples obtained from patients from consanguineous families with SCZ is important to make our results more meaningful. Furthermore, we plan to analyze genetic variants in updated ROHs by the next-generation sequencing technologies.

## Supporting Information

Table S1 Novel loci identified in this study.
(DOC)
