# Supplementary material for: Identification of Novel Schizophrenia Loci by Homozygosity Mapping Using DNA Microarray Analysis
Source: PLoS One. 2011 May 31;6(5):e20589. doi: 10.1371/journal.pone.0020589 (PMC3105082; doi:10.1371/journal.pone.0020589)
Supplement: Table S1 — Novel loci identified in this study. (DOC) [file pone.0020589.s001.doc]

**Table S1. Novel loci identified in this study.**

| **Chromosome** | **Start** | **End** | **Samples** | **# Samples** | **Length** | **Cytoband** |
| --- | --- | --- | --- | --- | --- | --- |
| 1 | 72280851 | 74696984 | h-1, a, g | 3 | 2416134 | 1p31.1 |
| 1 | 146258078 | 148749860 | h-1, h-2, a | 3 | 2491783 | 1q21.1-q21.2 |
| 1 | 178829362 | 182206937 | e, f, g | 3 | 3377576 | 1q25.3 |
| 3 | 42698831 | 45123678 | c, e, g | 3 | 2424848 | 3p22.1-p21.31 |
| 3 | 89718321 | 89834629 | h-1, a, c | 3 | 116309 | 3p11.1 |
| 3 | 89834629 | 95415799 | h-1, a, c, g | 4 | 5581171 | 3p11.1-q11.2 |
| 3 | 95415799 | 95419921 | h-1, c, g | 3 | 4123 | 3q11.2 |
| 3 | 166068269 | 168650842 | h-2, a, g | 3 | 2582574 | 3q26.1 |
| 4 | 61566 | 4235598 | c, f, g | 3 | 4174033 | 4p16.3-p16.2 |
| 4 | 34570250 | 41061846 | a, c, g | 3 | 6491597 | 4p15.1-p13 |
| 4 | 48695607 | 52991067 | b, c, g | 3 | 4295461 | 4p12-q12 |
| 5 | 45437574 | 49631829 | h-1, h-2, d | 3 | 4194256 | 5p12-q11.1 |
| 5 | 94414226 | 98552712 | b, f, g | 3 | 4138487 | 5q15-q21.1 |
| 5 | 98552712 | 98906572 | a, b, f, g | 4 | 353861 | 5q21.1 |
| 5 | 98906572 | 98959050 | a, b, f | 3 | 52479 | 5q21.1 |
| 5 | 98959050 | 100277241 | a, b, f, g | 4 | 1318192 | 5q21.1 |
| 5 | 100277241 | 100373206 | a, b, g | 3 | 95966 | 5q21.1 |
| 5 | 100373206 | 102994991 | a, b, f, g | 4 | 2621786 | 5q21.1-q21.2 |
| 5 | 102994991 | 103056748 | a, f, g | 3 | 61758 | 5q21.2 |
| 5 | 103056748 | 103720924 | a, b, f, g | 4 | 664177 | 5q21.2 |
| 5 | 103720924 | 104226158 | a, b, f | 3 | 505235 | 5q21.2 |
| 5 | 104226158 | 105941993 | a, b, f, g | 4 | 1715836 | 5q21.2-q21.3 |
| 5 | 105941993 | 106780236 | h-1, a, b, f, g | 5 | 838244 | 5q21.3 |
| 5 | 106780236 | 106807591 | h-1, a, b, f | 4 | 27356 | 5q21.3 |
| 5 | 106807591 | 108046277 | h-1, a, b, f, g | 5 | 1238687 | 5q21.3 |
| 5 | 108046277 | 108215388 | h-1, a, b, g | 4 | 169112 | 5q21.3 |
| 5 | 108215388 | 109448930 | h-1, a, b, f, g | 5 | 1233543 | 5q21.3 |
| 5 | 109448930 | 112559231 | h-1, a, f, g | 4 | 3110302 | 5q21.3-q22.2 |
| 5 | 112559231 | 114820097 | h-1, a, f | 3 | 2260867 | 5q22.2-q22.3 |
| 5 | 117360252 | 120214932 | h-1, h-2, f | 3 | 2854681 | 5q23.1 |
| 5 | 120214932 | 122586267 | h-1, h-2, f, g | 4 | 2371336 | 5q21.1-q23.2 |
| 5 | 122586267 | 122644111 | h-1, f, g | 3 | 57845 | 5q23.2 |
| 5 | 129002141 | 132519461 | h-1, c, f | 3 | 3517321 | 5q23.3-q31.1 |
| 6 | 58809855 | 62640811 | a, b, e | 3 | 3830957 | 6p11.1-q11.1 |
| 7 | 57492538 | 57594442 | h-2, b, f | 3 | 101905 | 7p11.1 |
| 7 | 57594442 | 62282881 | h-1, h-2, b, f | 4 | 4688440 | 7p11.1-q11.21 |
| 7 | 62282881 | 63072712 | h-2, b, f | 3 | 789832 | 7q11.21 |
| 7 | 63859252 | 66437018 | h-2, a, f | 3 | 2577767 | 7q11.21-q11.22 |
| 7 | 67990654 | 68045677 | h-2, c, f | 3 | 55024 | 7q11.22 |
| 8 | 42593162 | 42737870 | h-1, d, e, g | 4 | 144709 | 8p11.21 |
| 8 | 42737870 | 42744820 | h-1, b, d, e, g | 5 | 6951 | 8p11.21 |
| 8 | 42744820 | 47043376 | h-1, b, c, d, e, g | 6 | 4298557 | 8p11.21-q11.1 |
| 8 | 111137054 | 113520838 | b, d, f | 3 | 2383785 | 8q23.2-q23.3 |
| 8 | 129121122 | 131617749 | h-1, h-2, b | 3 | 2496628 | 8q24.21-q24.22 |
| 8 | 132434559 | 139244531 | h-1, h-2, b | 3 | 6809973 | 8q24.22-q24.23 |
| 9 | 38728489 | 70346371 | b, e, f | 3 | 31617883 | 9p13.1-q13 |
| 9 | 90729879 | 91937158 | h-2, b, g | 3 | 1207280 | 9q22.1-q22.2 |
| 9 | 118069545 | 120364847 | h-2, b, g | 3 | 2295303 | 9q33.1 |
| 10 | 37363792 | 37599485 | h-1, h-2, e | 3 | 235694 | 10p11.21 |
| 10 | 37599485 | 37874740 | h-1, h-2, e, g | 4 | 275256 | 10p11.21 |
| 10 | 37874740 | 42217616 | h-1, h-2, c, e, g | 5 | 4342877 | 10p11.21-q11.21 |
| 10 | 42217616 | 42519464 | c, e, g | 3 | 301849 | 10q11.21 |
| 10 | 52445050 | 54746707 | a, b, e | 3 | 2301658 | 10q11.23-q21.1 |
| 11 | 47932370 | 48614746 | a, d, g | 3 | 682377 | 11p11.2 |
| 11 | 49107686 | 51245914 | a, b, d | 3 | 2138229 | 11p11.12 |
| 11 | 51245914 | 55034193 | a, b, c, d | 4 | 3788280 | 11p11.12-q11 |
| 12 | 33982292 | 36255461 | h-1, h-2, a, d | 4 | 2273170 | 12p11.1-q11 |
| 12 | 36255461 | 36796428 | h-1, a, d | 3 | 540968 | 12q11-q12 |
| 12 | 96174379 | 98039484 | h-1, b, g | 3 | 1865106 | 12q23.1 |
| 12 | 101150849 | 117675866 | b, c, g | 3 | 16525018 | 12q23.2-q24.23 |
| 12 | 117675866 | 117796429 | a, b, c, g | 4 | 120564 | 12q24.23 |
| 12 | 117796429 | 124565483 | a, b, c | 3 | 6769055 | 12q24.23-q24.32 |
| 13 | 35366458 | 43580724 | h-1, h-2, g | 3 | 8214267 | 13q13.3-q14.11 |
| 16 | 28924029 | 29606107 | h-1, h-2, c | 3 | 682079 | 16p11.2 |
| 16 | 29606107 | 29657036 | h-1, h-2, c, f | 4 | 50930 | 16p11.2 |
| 16 | 29657036 | 29680943 | h-1, h-2, c, d, f | 5 | 23908 | 16p11.2 |
| 16 | 29680943 | 31277953 | h-1, h-2, b, c, d, f | 6 | 1597011 | 16p11.2 |
| 16 | 31277953 | 31567928 | h-1, b, c, d, f | 5 | 289976 | 16p11.2 |
| 16 | 31567928 | 32370399 | h-1, a, b, c, d, f | 6 | 802472 | 16p11.2 |
| 16 | 32370399 | 34454657 | h-1, a, b, c, d, f, g | 7 | 2084259 | 16p11.2-p11.1 |
| 16 | 34454657 | 34467305 | h-1, a, b, d, f, g | 6 | 12649 | 16p11.1 |
| 16 | 34467305 | 34647935 | h-1, h-2, a, b, c, d, f, g | 8 | 180631 | 16p11.1 |
| 16 | 34647935 | 45122807 | h-1, h-2, a, c, d, f, g | 7 | 10474873 | 16p11.1-q11.2 |
| 16 | 45122807 | 47094922 | h-1, h-2, a, b, c, d, f, g | 8 | 1972116 | 16q11.2-q12.1 |
| 16 | 47094922 | 47118442 | h-1, a, b, d, f, g | 6 | 23521 | 16q12.1 |
| 16 | 47118442 | 47320826 | h-1, a, b, d, f | 5 | 202385 | 16q12.1 |
| 16 | 47320826 | 47325025 | h-1, b, d, f | 4 | 4200 | 16q12.1 |
| 16 | 47325025 | 47418431 | h-1, d, f | 3 | 93407 | 16q12.1 |
| 16 | 65072463 | 67246920 | h-1, b, f | 3 | 2174458 | 16q21-q22.1 |
| 17 | 29659797 | 32811528 | h-1, h-2, a | 3 | 3151732 | 17q12 |
| 19 | 22694550 | 23279090 | h-1, d, e | 3 | 584541 | 19p12 |
| 19 | 23279090 | 23534946 | h-1, d, e, g | 4 | 255857 | 19p12 |
| 19 | 23534946 | 23748913 | h-1, a, d, e, g | 5 | 213968 | 19p12 |
| 19 | 23748913 | 23750719 | h-1, a, d, e, f, g | 6 | 1807 | 19p12 |
| 19 | 23750719 | 33106416 | h-1, a, b, d, e, f, g | 7 | 9355698 | 19p12-q12 |
| 19 | 33106416 | 33141651 | h-1, a, b, d, f, g | 6 | 35236 | 19q12 |
| 19 | 33141651 | 33152104 | h-1, b, f, g | 4 | 10454 | 19q12 |
| 19 | 33152104 | 33243081 | h-1, b, f | 3 | 90978 | 19q12 |
| 19 | 37676724 | 40349191 | h-1, h-2, a | 3 | 2672468 | 19q13.11-q13.12 |
| 20 | 24664288 | 25125805 | h-1, c, d | 3 | 461518 | 20p11.21 |
| 20 | 25125805 | 25144655 | h-1, b, c, d | 4 | 18851 | 20p11.21 |
| 20 | 25144655 | 25938002 | h-1, b, c, d, e | 5 | 793348 | 20p11.21-p11.1 |
| 20 | 25938002 | 26173145 | h-1, a, b, c, d, e | 6 | 235144 | 20p11.1 |
| 20 | 26173145 | 29309964 | h-1, a, b, c, d, e, f | 7 | 3136820 | 20p11.1-q11.21 |
| 20 | 29309964 | 29936849 | h-1, a, b, c, e, f | 6 | 626886 | 20q11.21 |
| 20 | 29936849 | 30491788 | h-1, a, c, e, f | 5 | 554940 | 20q11.21 |
| 20 | 30491788 | 30911029 | h-1, a, c, e | 4 | 419242 | 20q11.21 |
| 20 | 30911029 | 30916617 | h-1, a, c | 3 | 5589 | 20q11.21 |
| 21 | 19821557 | 20188026 | h-1, h-2, g | 3 | 366470 | 21q21.1 |

Overlapping segments shared among 3 or more unrelated individuals. In the column for the number of samples, the number of individuals who shared the region is shown, for example, if the number 3 is indicated, that means that 3 individuals shared the region.
